# Supplementary material for: Systematic review: progress in EEG-based speech imagery brain-computer interface decoding and encoding research
Source: PeerJ Comput Sci. 2025 Jun 23;11:e2938. doi: 10.7717/peerj-cs.2938 (PMC12453656; doi:10.7717/peerj-cs.2938)
Supplement: Supplemental Information 2 [file peerj-cs-11-2938-s002.docx]

**Summary of references**

1. Wolpaw, J. R., Millan, J. D. R., & Ramsey, N. F. (2020). Brain-computer interfaces: definitions and principles. Handbook of clinical neurology, 168, 15-23.
2. Liu, Y., Gong, A., Ding, P., Zhao, L., Qian, Q. I. A. N., Zhou, J., Su, L. & Fu, Y. (2022). Key technology of brain-computer interaction based on speech imagery. Sheng wu yi xue Gong Cheng xue za zhi= Journal of Biomedical Engineering= Shengwu Yixue Gongchengxue Zazhi, 39(3), 596-611.
3. Lee, S. H., Lee, M., & Lee, S. W. (2020). Neural decoding of imagined speech and visual imagery as intuitive paradigms for BCI communication. IEEE Transactions on Neural Systems and Rehabilitation Engineering, 28(12), 2647-2659.
4. Pawar, D., & Dhage, S. (2022, April). Imagined speech classification using EEG based brain-computer interface. In 2022 IEEE 11th International Conference on Communication Systems and Network Technologies (CSNT) (pp. 662-666). IEEE.
5. Qureshi, M. N. I., Min, B., Park, H. J., Cho, D., Choi, W., & Lee, B. (2017). Multiclass classification of word imagination speech with hybrid connectivity features. IEEE Transactions on Biomedical Engineering, 65(10), 2168-2177.
6. Lu, L., Han, M., Zou, G., Zheng, L., & Gao, J. H. (2023). Common and distinct neural representations of imagined and perceived speech. Cerebral Cortex, 33(10), 6486-6493.
7. Beyeler, A., Namburi, P., Glober, G.F., Simonnet, C., Calhoon, G.G., Conyers, G.F., Luck, R., Wildes, C.P. & Tye, K.M. (2016). Divergent routing of positive and negative information from the amygdala during memory retrieval. Neuron, 90(2), pp.348-361.
8. Peña Serrano, N., Jaimes-Reátegui, R., & Pisarchik, A. N. (2024). Hypergraph of functional connectivity based on event-related coherence: Magnetoencephalography data analysis. Applied Sciences, 14(6), 2343.
9. Hurlburt, R. T., Alderson-Day, B., Kühn, S., & Fernyhough, C. (2016). Exploring the ecological validity of thinking on demand: Neural correlates of elicited vs. spontaneously occurring inner speech. PLoS one, 11(2), e0147932.
10. Rekrut, M., Selim, A. M., & Krüger, A. (2022, October). Improving silent speech bci training procedures through transfer from overt to silent speech. In 2022 IEEE International Conference on Systems, Man, and Cybernetics (SMC) (pp. 2650-2656). IEEE.
11. Chen, F., & Pan, C. (2020). A review on brain-computer interface technologies for speech imagery[J]. Journal of Signal Processing, 36(6).
12. Schultz, T., Wand, M., Hueber, T., Krusienski, D. J., Herff, C., & Brumberg, J. S. (2017). Biosignal-based spoken communication: A survey. IEEE/ACM Transactions on Audio, Speech, and Language Processing, 25(12), 2257-2271.
13. Cooney, C., Folli, R., & Coyle, D. (2018). Neurolinguistics research advancing development of a direct-speech brain-computer interface. IScience, 8, 103-125.
14. Alzahrani, S., Banjar, H., & Mirza, R. (2024). Systematic Review of EEG-Based Imagined Speech Classification Methods. Sensors, 24(24), 8168.
15. Lopez-Bernal, D., Balderas, D., Ponce, P., & Molina, A. (2022). A state-of-the-art review of EEG-based imagined speech decoding. Frontiers in human neuroscience, 16, 867281.
16. Rahman, N., Khan, D. M., Masroor, K., Arshad, M., Rafiq, A., & Fahim, S. M. (2024). Advances in brain-computer interface for decoding speech imagery from EEG signals: a systematic review. Cognitive Neurodynamics, 1-19.
17. Zhang, L., Zhou, Y., Gong, P., & Zhang, D. (2024). Speech imagery decoding using EEG signals and deep learning: A survey. IEEE Transactions on Cognitive and Developmental Systems.
18. Panachakel, J. T., & Ramakrishnan, A. G. (2021). Decoding covert speech from EEG-a comprehensive review.  Frontiers in Neuroscience, 15, 642251.
19. Kaongoen, N., Choi, J., Choi, J. W., Kwon, H., Hwang, C., Hwang, G., Kim, B. H., & Jo, S. (2023). The future of wearable EEG: A review of ear-EEG technology and its applications. Journal of neural engineering.
20. He, B., Astolfi, L., Valdés-Sosa, P. A., Marinazzo, D., Palva, S. O., Bénar, C. G., Michel, C. M., & Koenig, T. (2019). Electrophysiological brain connectivity: theory and implementation. IEEE transactions on biomedical engineering, 66(7), 2115-2137.
21. Kristensen, A. B., Subhi, Y., & Puthusserypady, S. (2020). Vocal imagery vs intention: viability of vocal-based EEG-BCI paradigms. IEEE Transactions on Neural Systems and Rehabilitation Engineering, 28(8), 1750-1759.
22. Page MJ, McKenzie JE, Bossuyt PM, Boutron I, Hoffmann TC, Mulrow CD, Shamseer L, Tetzlaff JM, Akl EA, Brennan SE, Chou R, Glanville J, Grimshaw JM, Hróbjartsson A, Lalu MM, Li T, Loder EW, Mayo-Wilson E, McDonald S, McGuinness LA, Stewart LA, Thomas J, Tricco AC, Welch VA, Whiting P, Moher D (2021) The PRISMA 2020 statement: an updated guideline for reporting systematic reviews. BMJ 372
23. Jäncke, L., Liem, F., & Merillat, S. (2021). Are language skills related to structural features in Broca's and Wernicke's area?. European Journal of Neuroscience, 53(4), 1124-1135.
24. Ono, Y., Zhang, X., Noah, J. A., Dravida, S., & Hirsch, J. (2022). Bidirectional connectivity between Broca's area and Wernicke's area during interactive verbal communication. Brain connectivity, 12(3), 210-222.
25. Sandhya, C., Srinidhi, G., Vaishali, R., Visali, M., & Kavitha, A. (2015, July). Analysis of speech imagery using brain connectivity estimators. In 2015 IEEE 14th International Conference on Cognitive Informatics & Cognitive Computing (ICCI* CC) (pp. 352-359). IEEE.
26. Chengaiyan, S., & Anandhan, K. (2015). Analysis of speech imagery using functional and effective EEG based brain connectivity parameters. International Journal of Cognitive Informatics and Natural Intelligence (IJCINI), 9(4), 33-48.
27. Chengaiyan, S., Balathayil, D., Anandan, K., & Thomas, C. B. (2018). Effect of power and phase synchronization in multi-trial speech imagery. International Journal of Software Science and Computational Intelligence (IJSSCI), 10(4), 44-61.
28. Bisla, M., & Anand, R. S. (2023, August). Analysis of Imagined Speech Characteristics using Phase-based Connectivity Measures. In 2023 IEEE 13th International Conference on Control System, Computing and Engineering (ICCSCE) (pp. 68-73). IEEE.
29. Chengaiyan, S., & Anandan, K. (2022). Effect of functional and effective brain connectivity in identifying vowels from articulation imagery procedures. Cognitive Processing, 23(4), 593-618.
30. Park, S. M., Yeom, H. G., & Sim, K. B. (2021). User State Classification Based on Functional Brain Connectivity Using a Convolutional Neural Network. Electronics, 10(10), 1158.
31. Belaoucha, B., & Papadopoulo, T. (2020). Structural connectivity to reconstruct brain activation and effective connectivity between brain regions. Journal of Neural Engineering, 17(3), 035006.
32. Panachakel, J. T., & Ramakrishnan, A. G. (2021, November). Classification of phonological categories in imagined speech using phase synchronization measure. In 2021 43rd Annual International Conference of the IEEE Engineering in Medicine & Biology Society (EMBC) (pp. 2226-2229). IEEE.
33. Chengaiyan, S., Retnapandian, A. S., & Anandan, K. (2020). Identification of vowels in consonant–vowel–consonant words from speech imagery based EEG signals. Cognitive Neurodynamics, 14(1), 1-19.
34. Ahn, H. J., Lee, D. H., Jeong, J. H., & Lee, S. W. (2022). Multiscale convolutional transformer for EEG classification of mental imagery in different modalities. IEEE Transactions on Neural Systems and Rehabilitation Engineering, 31, 646-656.
35. Tai, P., Ding, P., Wang, F., Gong, A., Li, T., Zhao, L., Su, L., & Fu, Y. (2024). Brain-computer interface paradigms and neural coding. Frontiers in Neuroscience, 17, 1345961.
36. DaSalla, C. S., Kambara, H., Sato, M., & Koike, Y. (2009). Single-trial classification of vowel speech imagery using common spatial patterns. Neural networks, 22(9), 1334-1339.
37. Min, B., Kim, J., Park, H. J., & Lee, B. (2016). Vowel imagery decoding toward silent speech BCI using extreme learning machine with electroencephalogram. BioMed research international, 2016(1), 2618265.
38. Mahapatra, N. C., & Bhuyan, P. (2022). Multiclass classification of imagined speech vowels and words of electroencephalography signals using deep learning. Advances in Human‐Computer Interaction, 2022(1), 1374880.
39. Sandhya, C., Divya, B., Kavitha, A., & Bobby, T. C. (2018, July). Influence of Relative Power in Multi-Trial Speech Imagery. In 2018 IEEE 17th International Conference on Cognitive Informatics & Cognitive Computing (ICCI* CC) (pp. 431-439). IEEE.
40. Wang, L., Zhang, X., Zhong, X., & Fan, Z. (2016). Improvement of mental tasks with relevant speech imagery for brain-computer interfaces. Measurement, 91, 201-209.
41. Li, H., & Chen, F. (2020). Classify imaginary mandarin tones with cortical eeg signals. In INTERSPEECH (pp. 4896-4900).
42. Zhang, X., Li, H., & Chen, F. (2020, July). EEG-based classification of imaginary Mandarin tones. In 2020 42nd Annual International Conference of the IEEE Engineering in Medicine & Biology Society (EMBC) (pp. 3889-3892). IEEE.
43. Pan, H., Li, Z., Tian, C., Wang, L., Fu, Y., Qin, X., & Liu, F. (2023). The LightGBM-based classification algorithm for Chinese characters speech imagery BCI system. Cognitive Neurodynamics, 17(2), 373-384.
44. Tsukahara, A., Yamada, M., Tanaka, K., & Uchikawa, Y. (2019). An examination of EEG frequency components related to speech imagery and its identification. IEEJ Transactions on Electronics, Information and Systems, 139(5), 588-595.
45. Kaongoen, N., Choi, J., & Jo, S. (2021). Speech-imagery-based brain-computer interface system using ear-EEG. Journal of neural engineering, 18(1), 016023.
46. Borirakarawin, M., & Punsawad, Y. (2023). Hybrid Brain–Computer Interface System Using Auditory Stimulation and Speech Imagination Paradigms for Assistive Technology. IEEE Access, 11, 53079-53090.
47. Hernandez-Galvan, A., Ramirez-Alonso, G., & Ramirez-Quintana, J. (2023). A prototypical network for few-shot recognition of speech imagery data. Biomedical Signal Processing and Control, 86, 105154.
48. Naebi, A., & Feng, Z. (2023). The Performance of a Lip-Sync Imagery Model, New Combinations of Signals, a Supplemental Bond Graph Classifier, and Deep Formula Detection as an Extraction and Root Classifier for Electroencephalograms and Brain–Computer Interfaces.  Applied Sciences, 13(21), 11787.
49. Xu, L., Xu, M., Jung, T. P., & Ming, D. (2021). Review of brain encoding and decoding mechanisms for EEG-based brain–computer interface. Cognitive neurodynamics, 15, 569-584.
50. Jahangiri, A., & Sepulveda, F. (2017, July). The contribution of different frequency bands in class separability of covert speech tasks for BCIs. In 2017 39th Annual International Conference of the IEEE Engineering in Medicine and Biology Society (EMBC) (pp. 2093-2096). IEEE.
51. Moattari, M., Parnianpour, P., & Moradi, M. H. (2017, May). Independent component analysis approach using higher orders of Non-Gaussianity. In 2017 Iranian Conference on Electrical Engineering (ICEE) (pp. 49-54). IEEE.
52. Nitta, T., Horikawa, J., Iribe, Y., Taguchi, R., Katsurada, K., Shinohara, S., & Kawai, G. (2023). Linguistic representation of vowels in speech imagery EEG. Frontiers in Human Neuroscience, 17, 1163578.
53. Sree, R. A., Kavitha, A., & Divya, B. (2023). Optimization of pre-processing routines in speech imagery-based EEG signals. Journal of Mechanics in Medicine and Biology, 23(06), 2340032.
54. Wang, L., Yan, Z., Liu, Y., & Hu, L. (2021). Analysis and application of functional connectivity in synchronic hybrid mental tasks for brain-computer interface. Measurement, 186, 110116.
55. Huang, X., Wang, L., Ren, L., & Zhan, Q. (2022, April). EEG Signals Recognition of Active Brain-Computer Interface Based on Sequential Encoding. In 2022 7th International Conference on Intelligent Computing and Signal Processing (ICSP) (pp. 1821-1826). IEEE.
56. Alizadeh, D., & Omranpour, H. (2023). EM-CSP: an efficient multiclass common spatial pattern feature method for speech imagery EEG signals recognition. Biomedical Signal Processing and Control, 84, 104933.
57. Cooney, C., Folli, R., & Coyle, D. (2018, June). Mel frequency cepstral coefficients enhance imagined speech decoding accuracy from EEG. In 2018 29th Irish Signals and Systems Conference (ISSC) (pp. 1-7). IEEE.
58. Martin, S., Brunner, P., Iturrate, I., Millán, J. D. R., Schalk, G., Knight, R. T., & Pasley, B. N. (2016). Word pair classification during imagined speech using direct brain recordings. Scientific reports, 6(1), 25803.
59. Wu, H., & Chen, F. (2020, December). A temporal envelope-based speech reconstruction approach with EEG signals during speech imagery. In 2020 Asia-Pacific Signal and Information Processing Association Annual Summit and Conference (APSIPA ASC) (pp. 894-899). IEEE.
60. Idrees, B. M., & Farooq, O. (2016, March). EEG based vowel classification during speech imagery. In 2016 3rd International Conference on Computing for Sustainable Global Development (INDIACom) (pp. 1130-1134). IEEE.
61. Idrees, B. M., & Farooq, O. (2016, February). Vowel classification using wavelet decomposition during speech imagery. In 2016 3rd International Conference on Signal Processing and Integrated Networks (SPIN) (pp. 636-640). IEEE.
62. Saji, A., Shanir, P. P., Sha, A. A., & Kunju, N. (2020, April). Analysis of EEG for classification vowel sounds. In AIP Conference Proceedings (Vol. 2222, No. 1). AIP Publishing.
63. Pan, C., Liu, H., Zheng, D., & Chen, F. (2022, July). Neural Entrainment to Rhythms of Imagined Syllables. In 2022 44th Annual International Conference of the IEEE Engineering in Medicine & Biology Society (EMBC) (pp. 4040-4043). IEEE.
64. Kamble, A., Ghare, P. H., & Kumar, V. (2022). Deep-learning-based BCI for automatic imagined speech recognition using SPWVD. IEEE Transactions on Instrumentation and Measurement, 72, 1-10.
65. Wang, J., & Wang, L. (2022, April). Parallel Convolutional Neural Network Based on Multi-Band Brain Networks for EEG Classification. In 2022 5th International Conference on Advanced Electronic Materials, Computers and Software Engineering (AEMCSE) (pp. 49-53). IEEE.
66. Ramirez-Quintana, J. A., Macias-Macias, J. M., Ramirez-Alonso, G., Chacon-Murguia, M. I., & Corral-Martinez, L. F. (2023). A novel deep capsule neural network for vowel imagery patterns from EEG signals. Biomedical Signal Processing and Control, 81, 104500.
67. Retnapandian, A. S., & Anandan, K. (2023). Phoneme-based Imagined Vowel Identification from Electroencephalographic Sub-Band Oscillations during Speech Imagery Procedures.
68. Macías-Macías, J. M., Ramírez-Quintana, J. A., Ramírez-Alonso, G., & Chacón-Murguía, M. I. (2020, November). Deep learning networks for vowel speech imagery. In 2020 17th International Conference on Electrical Engineering, Computing Science and Automatic Control (CCE) (pp. 1-6). IEEE.
69. Sikdar, D., Roy, R., Bakshi, K., & Mahadevappa, M. (2018, July). Multifractal Analysis of Speech Imagery of IPA Vowels. In 2018 40th Annual International Conference of the IEEE Engineering in Medicine and Biology Society (EMBC) (pp. 1-4). IEEE.
70. Sikdar, D., Roy, R., & Mahadevappa, M. (2018, November). Chaos Analysis of Speech Imagery of IPA Vowels. In International Conference on Intelligent Human Computer Interaction (pp. 101-110). Cham: Springer International Publishing.
71. Nguyen, C. H., Karavas, G. K., & Artemiadis, P. (2017). Inferring imagined speech using EEG signals: a new approach using Riemannian manifold features. Journal of neural engineering, 15(1), 016002.
72. Kalaganis, F. P., Georgiadis, K., Oikonomou, V. P., Nikolopoulos, S., Laskaris, N. A., & Kompatsiaris, I. (2023, August). Exploiting Approximate Joint Diagonalization for Covariance Estimation in Imagined Speech Decoding. In International Conference on Brain Informatics (pp. 409-419). Cham: Springer Nature Switzerland.
73. Saha, P., & Fels, S. (2019, July). Hierarchical deep feature learning for decoding imagined speech from EEG. In Proceedings of the AAAI Conference on Artificial Intelligence (Vol. 33, No. 01, pp. 10019-10020).
74. Cai, G., Zhang, F., Yang, B., Huang, S., & Ma, T. (2024). Manifold Learning-based Common Spatial Pattern for EEG Signal Classification. IEEE Journal of Biomedical and Health Informatics.
75. Rezvani, S., Pourpanah, F., Lim, C. P., & Wu, Q. J. (2024). Methods for class-imbalanced learning with support vector machines: a review and an empirical evaluation. Soft Computing, 1-22.
76. Ji, H., Liu, X., Zhang, J., & Liu, L. (2024). Spatial Localization of a Transformer Robot Based on Ultrasonic Signal Wavelet Decomposition and PHAT-β-γ Generalized Cross Correlation. Sensors, 24(5), 1440.
77. Luo, H., Guo, Z., Chen, W., Gao, X., & Fan, J. (2024). Structural health assessment of historical buildings with nonuniform FE analysis and modified covariance matrix adaptation evaluation strategy (CMAES) updating. Automation in Construction, 166, 105618.
78. Khatti, J., & Grover, K. S. (2024). Prediction of uniaxial strength of rocks using relevance vector machine improved with dual kernels and metaheuristic algorithms. Rock Mechanics and Rock Engineering, 1-32.
79. Wei, X., Lang, P., Li, J., Feng, K., & Zhan, Y. (2024). A hybrid optimization method based on extreme learning machine aided factor graph for INS/GPS information fusion during GPS outages. Aerospace Science and Technology, 109326.
80. Liang, Y., Tan, J., Xie, Z., Chen, Z., Lin, D., & Yang, Z. (2023). Research on Convolutional Neural Network Inference Acceleration and Performance Optimization for Edge Intelligence. Sensors, 24(1), 240.
81. Aboussaleh, I., Riffi, J., Fazazy, K. E., Mahraz, A. M., & Tairi, H. (2024). STCPU-Net: advanced U-shaped deep learning architecture based on Swin transformers and capsule neural network for brain tumor segmentation. Neural Computing and Applications, 36(30), 18549-18565.
82. Li, H., Qiao, R., Yu, P., Li, H., & Tan, M. (2024). CTHD-Net: CNN-Transformer hybrid dehazing network via residual global attention and gated boosting strategy. Journal of Visual Communication and Image Representation, 99, 104066.
83. Zhao, S., & Rudzicz, F. (2015, April). Classifying phonological categories in imagined and articulated speech. In 2015 IEEE international conference on acoustics, speech and signal processing (ICASSP) (pp. 992-996). IEEE.
84. Mou, X., He, C., Tan, L., Yu, J., Liang, H., Zhang, J., Tian, Y., Yang, Y., Xu, T., Wang, Q., Cao, M., Chen, Z., Hu, C., Wang, X., Liu, Q. & Wu, H. (2024). ChineseEEG: A Chinese linguistic corpora EEG dataset for semantic alignment and neural decoding. Scientific Data, 11(1), 550.
85. Agarwal, P., Kale, R. K., Kumar, M., & Kumar, S. (2020, February). Silent speech classification based upon various feature extraction methods. In 2020 7th International Conference on Signal Processing and Integrated Networks (SPIN) (pp. 16-20). IEEE.
86. Rostami, M., & Moradi, M. H. (2015, November). Evidential multi-band common spatial pattern in brain computer interface. In 2015 22nd Iranian Conference on Biomedical Engineering (ICBME) (pp. 16-20). IEEE.
87. Lee, B. H., Kwon, B. H., Lee, D. Y., & Jeong, J. H. (2021, February). Speech imagery classification using length-wise training based on deep learning. In 2021 9th International Winter Conference on Brain-Computer Interface (BCI) (pp. 1-5). IEEE.
88. Kwon, H., Choi, J., Choi, J. W., & Jo, S. (2023, February). Subject-Aware User State Classification with Deep Learning Models: An Exploratory Study. In 2023 11th International Winter Conference on Brain-Computer Interface (BCI) (pp. 1-4). IEEE.
89. Gomez, A. N., Ren, M., Urtasun, R., & Grosse, R. B. (2017). The reversible residual network: Backpropagation without storing activations. Advances in neural information processing systems, 30.
90. Vorontsov, E., Trabelsi, C., Kadoury, S., & Pal, C. (2017, July). On orthogonality and learning recurrent networks with long term dependencies. In International Conference on Machine Learning (pp. 3570-3578). PMLR.
91. Jeong, J. H., Cho, J. H., Lee, B. H., & Lee, S. W. (2022). Real-time deep neurolinguistic learning enhances noninvasive neural language decoding for brain–machine interaction. IEEE Transactions on Cybernetics, 53(12), 7469-7482.
92. Macías-Macías, J. M., Ramírez-Quintana, J. A., Chacón-Murguía, M. I., Torres-García, A. A., & Corral-Martínez, L. F. (2023). Interpretation of a deep analysis of speech imagery features extracted by a capsule neural network. Computers in Biology and Medicine, 159, 106909.
93. Watanabe, H., Tanaka, H., Sakti, S., & Nakamura, S. (2020). Synchronization between overt speech envelope and EEG oscillations during imagined speech. Neuroscience research, 153, 48-55.
94. Islam, M. M., & Shuvo, M. M. H. (2019, September). DenseNet based speech imagery EEG signal classification using Gramian Angular Field. In 2019 5th International Conference on Advances in Electrical Engineering (ICAEE) (pp. 149-154). IEEE.
95. Panachakel, J. T., & Ganesan, R. A. (2021). Decoding imagined speech from EEG using transfer learning. IEEE Access, 9, 135371-135383.
96. Kim, H. J., Lee, M. H., & Lee, M. (2020, February). A BCI based smart home system combined with event-related potentials and speech imagery task. In 2020 8th International Winter Conference on Brain-Computer Interface (BCI) (pp. 1-6). IEEE.
97. Lee, D. H., Jeong, J. H., Ahn, H. J., & Lee, S. W. (2021, February). Design of an EEG-based drone swarm control system using endogenous BCI paradigms. In 2021 9th International Winter Conference on Brain-Computer Interface (BCI) (pp. 1-5). IEEE.
98. Jafferson, A. J., Ponnusamy, V., Jovic, J., & Trajanovic, M. (2021). An iot based cloud EEG signal analytic framework for thought to text mapping. IEIE Transactions on Smart Processing & Computing, 10(3), 183-188.
99. Zhao, Y., Liu, Y., & Gao, Y. (2021). Analysis and classification of speech imagery EEG based on Chinese initials. Journal of Beijing Institute of Technology, 30(zk), 44-51.
100. Pan, H., Wang, Y., Li, Z., Chu, X., Teng, B., & Gao, H. (2024). A complete scheme for multi-character classification using EEG signals from speech imagery. IEEE Transactions on Biomedical Engineering.
101. Wang, L., Huang, W., Yang, Z., Hu, X., & Zhang, C. (2020). A method from offline analysis to online training for the brain-computer interface based on motor imagery and speech imagery. Biomedical Signal Processing and Control, 62, 102100.
102. Sereshkeh, A. R., Trott, R., Bricout, A., & Chau, T. (2017). EEG classification of covert speech using regularized neural networks. IEEE/ACM Transactions on Audio, Speech, and Language Processing, 25(12), 2292-2300.
103. Panachakel, J. T., & Ramakrishnan, A. G. (2022, August). DCLL—A Deep Network for Possible Real-Time Decoding of Imagined Words. In International Symposium on Intelligent Informatics (pp. 3-12). Singapore: Springer Nature Singapore.
104. Zhang, X., Yao, L., Sheng, Q. Z., Kanhere, S. S., Gu, T., & Zhang, D. (2018, March). Converting your thoughts to texts: Enabling brain typing via deep feature learning of eeg signals. In 2018 IEEE international conference on pervasive computing and communications (PerCom) (pp. 1-10). IEEE.
105. Ali, S., Mumtaz, W., & Maqsood, A. (2023, May). EEG based thought-to-text translation via deep learning. In 2023 7th International Multi-Topic ICT Conference (IMTIC) (pp. 1-8). IEEE.
106. Park, H. J., & Lee, B. (2023). Multiclass classification of imagined speech EEG using noise-assisted multivariate empirical mode decomposition and multireceptive field convolutional neural network. Frontiers in human neuroscience, 17, 1186594.
107. He, M., Wu, Y., Li, Z., Wang, S., Li, W., Zhou, W., Rong, H. & Wang, J. (2023, May). HMT: an EEG signal classification method based on CNN architecture. In 2023 5th International Conference on Intelligent Control, Measurement and Signal Processing (ICMSP) (pp. 1015-1018). IEEE.
108. Zhang, H., Guo, Z., & Chen, F. (2023, July). The effects of different brain regions on fNIRS-based task-state detection in speech imagery. In 2023 45th Annual International Conference of the IEEE Engineering in Medicine & Biology Society (EMBC) (pp. 1-4). IEEE.
109. Wang, L., Liu, X., Liang, Z., Yang, Z., & Hu, X. (2019). Analysis and classification of hybrid BCI based on motor imagery and speech imagery. Measurement, 147, 106842.
110. Wang, L., Huang, X., Ren, L., & Zhan, Q. (2022). Signal analysis and classification of a novel active brain-computer interface based on four-category sequential coding. Biomedical Signal Processing and Control, 78, 103857.
111. Tong, J., Xing, Z., Wei, X., Yue, C., Dong, E., Du, S., Sun, Z., Solé-Casals, J. & Caiafa, C. F. (2023). Towards Improving Motor Imagery Brain–Computer Interface Using Multimodal Speech Imagery. Journal of Medical and Biological Engineering, 43(3), 216-226.
112. Silva, A. B., Liu, J. R., Metzger, S. L., Bhaya-Grossman, I., Dougherty, M. E., Seaton, M. P., Littlejohn, K. T., Tu-Chan, A., Ganguly, K., Moses, D. A., Chang, E. F.& Chang, E. F. (2024). A bilingual speech neuroprosthesis driven by cortical articulatory representations shared between languages. Nature Biomedical Engineering, 8(8), 977-991.
113. Wang, M. L., Shao, W., Hao, X. K., & Zhang, D. Q. (2023). Machine learning for brain imaging genomics methods: a review. Machine intelligence research, 20(1), 57-78.
114. Guo, Z., & Chen, F. (2022). Idle-state detection in motor imagery of articulation using early information: A functional Near-infrared spectroscopy study. Biomedical Signal Processing and Control, 72, 103369.
115. Vorreuther, A., Bastian, L., Benitez Andonegui, A., Evenblij, D., Riecke, L., Lührs, M., & Sorger, B. (2023). It takes two (seconds): decreasing encoding time for two-choice functional near-infrared spectroscopy brain–computer interface communication. Neurophotonics, 10(4), 045005-045005.
116. Rezazadeh Sereshkeh, A., Yousefi, R., Wong, A. T., Rudzicz, F., & Chau, T. (2019). Development of a ternary hybrid fNIRS-EEG brain–computer interface based on imagined speech. Brain-Computer Interfaces, 6(4), 128-140.
117. Wang, L., Hu, L., Wang, J., & Liang, D. (2021, October). A Personalized Channel Selection and Spatial filtering Model for Brain-Computer Interface. In Proceedings of the 2021 10th International Conference on Bioinformatics and Biomedical Science (pp. 100-105).
118. Nguyen, C. H., Karavas, G. K., & Artemiadis, P. (2019). Adaptive multi-degree of freedom Brain Computer Interface using online feedback: Towards novel methods and metrics of mutual adaptation between humans and machines for BCI. PloS one, 14(3), e0212620.
119. Saha, P., Fels, S., & Abdul-Mageed, M. (2019, May). Deep learning the EEG manifold for phonological categorization from active thoughts. In ICASSP 2019-2019 IEEE International Conference on Acoustics, Speech and Signal Processing (ICASSP) (pp. 2762-2766). IEEE.
120. Cui, W., Wang, X., Li, M., Pun, S. H., & Chen, F. (2023, November). A Study of Deep Learning Based Classification of Mandarin Vowels Using Spoken Speech EEG Signals. In 2023 IEEE International Conference on Signal Processing, Communications and Computing (ICSPCC) (pp. 1-5). IEEE.
121. Wu, S., Bhadra, K., Giraud, A. L., & Marchesotti, S. (2024). Adaptive LDA Classifier Enhances Real-Time Control of an EEG Brain–Computer Interface for Decoding Imagined Syllables. Brain Sciences, 14(3), 196.
122. Committee, B. C. (2022, July 6). 2020 International BCI Competition.
123. Li, M., Pun, S. H., & Chen, F. (2021, May). A preliminary study of classifying spoken vowels with EEG signals. In 2021 10th International IEEE/EMBS Conference on Neural Engineering (NER) (pp. 13-16). IEEE.
124. ATR brainliner “A platform for sharing time-aligned brain activity and behavioral data.(n.d.).", from (October 11, 2015)
125. Bhadra, K., Giraud, A. L., & Marchesotti, S. (2025). Learning to operate an imagined speech Brain-Computer Interface involves the spatial and frequency tuning of neural activity. Communications Biology, 8(1), 271.
126. Choi, J., Kaongoen, N., & Jo, S. (2022, February). Investigation on effect of speech imagery eeg data augmentation with actual speech. In 2022 10th International Winter Conference on Brain-Computer Interface (BCI) (pp. 1-5). IEEE.
127. Douibi, K., Le Bars, S., Lemontey, A., Nag, L., Balp, R., & Breda, G. (2021). Toward EEG-based BCI applications for industry 4.0: Challenges and possible applications. Frontiers in Human Neuroscience, 15, 705064.
128. Chengaiyan, S., Retnapandian, A. S., & Anandan, K. (2020). Identification of vowels in consonant–vowel–consonant words from speech imagery based EEG signals. Cognitive Neurodynamics, 14(1), 1-19.
129. Lin, Y., Liu, B., Liu, Z., & Gao, X. (2015). EEG gamma-band activity during audiovisual speech comprehension in different noise environments. Cognitive neurodynamics, 9, 389-398.
130. Mehta, B., Chawla, V. K., ParaKh, M., ParaKh, P., Bhandari, B., & Gurjar, A. S. (2015). EEG abnormalities in children with speech and language impairment. Journal of Clinical and Diagnostic Research: JCDR, 9(7), CC04.
131. Sandhya, C., Anandha Sree, R., & Kavitha, A. (2016). Analysis of speech imagery using consonant–vowel speech syllable pairs and brain connectivity estimators. In Second international conference on biomedical signals, systems, images, IIT Madras, India.
132. Sree, R. A., & Kavitha, A. (2017, March). Vowel classification from imagined speech using sub-band EEG frequencies and deep belief networks. In 2017 fourth international conference on signal processing, communication and networking (ICSCN) (pp. 1-4). IEEE.
133. Mohamed Selim, A., Rekrut, M., Barz, M., & Sonntag, D. (2024, June). Speech imagery bci training using game with a purpose. In Proceedings of the 2024 International Conference on Advanced Visual Interfaces (pp. 1-5).
134. García-Salinas, J. S., Villaseñor-Pineda, L., Reyes-García, C. A., & Torres-García, A. A. (2019). Transfer learning in imagined speech EEG-based BCIs. Biomedical Signal Processing and Control, 50, 151-157.
135. Lee, S. H., Lee, M., & Lee, S. W. (2019, November). EEG representations of spatial and temporal features in imagined speech and overt speech. In Asian Conference on Pattern Recognition (pp. 387-400). Cham: Springer International Publishing.
136. Mohanchandra, K., & Saha, S. (2016). A communication paradigm using subvocalized speech: translating brain signals into speech. Augmented Human Research, 1(1), 3.
137. Pawar, D., & Dhage, S. (2020). Multiclass covert speech classification using extreme learning machine. Biomedical engineering letters, 10(2), 217-226.
138. Pawar, D., & Dhage, S. (2023). EEG-based covert speech decoding using random rotation extreme learning machine ensemble for intuitive BCI communication. Biomedical Signal Processing and Control, 80, 104379.
139. Sereshkeh, A. R., Trott, R., Bricout, A., & Chau, T. (2017). Online EEG classification of covert speech for brain–computer interfacing. International journal of neural systems, 27(08), 1750033.
140. Arun, B., & Samanta, D. (2024, July). Impact of Fragmentation on Temporal Event Localization for Speech EEG. In 2024 International Conference on Signal Processing and Communications (SPCOM) (pp. 1-5). IEEE.
141. Lee, S. H., Lee, M., Jeong, J. H., & Lee, S. W. (2019, October). Towards an EEG-based intuitive BCI communication system using imagined speech and visual imagery. In 2019 IEEE international conference on systems, man and cybernetics (SMC) (pp. 4409-4414). IEEE.
142. Agarwal, P., & Kumar, S. (2022). Electroencephalography‐based imagined speech recognition using deep long short‐term memory network. ETRI Journal, 44(4), 672-685.
143. Rusnac, A. L., & Grigore, O. (2021, November). EEG preprocessing methods for BCI imagined speech signals. In 2021 International Conference on e-Health and Bioengineering (EHB) (pp. 1-4). IEEE.
144. Dekker, B., Schouten, A. C., & Scharenborg, O. (2023, June). DAIS: The Delft database of EEG recordings of dutch articulated and imagined speech. In ICASSP 2023-2023 IEEE International Conference on Acoustics, Speech and Signal Processing (ICASSP) (pp. 1-5). IEEE.
145. Hernández-Del-Toro, T., Reyes-García, C. A., & Villaseñor-Pineda, L. (2021). Toward asynchronous EEG-based BCI: Detecting imagined words segments in continuous EEG signals. Biomedical Signal Processing and Control, 65, 102351.
146. Lee, D. H., Kim, S. J., & Lee, K. W. (2022, February). Decoding high–level imagined speech using attention–based deep neural networks. In 2022 10th International Winter Conference on Brain-Computer Interface (BCI) (pp. 1-4). IEEE.
147. Lee, Y. E., & Lee, S. H. (2022, February). EEG-transformer: Self-attention from transformer architecture for decoding EEG of imagined speech. In 2022 10th International winter conference on brain-computer interface (BCI) (pp. 1-4). IEEE.
148. Proix, T., Delgado Saa, J., Christen, A., Martin, S., Pasley, B. N., Knight, R. T., Tian, X., Poeppel, D., Doyle, W. K., Devinsky, O., Arnal, L. H., Mégevand, P., & Giraud, A. L. (2022). Imagined speech can be decoded from low-and cross-frequency intracranial EEG features. Nature communications, 13(1), 48.
149. Saha, P., Abdul-Mageed, M., & Fels, S. (2019). Speak your mind! towards imagined speech recognition with hierarchical deep learning. arxiv preprint arxiv:1904.05746.
150. Tong, J., Xing, Z., Wei, X., Yue, C., Dong, E., Du, S., ... & Caiafa, C. F. (2023). Towards improving motor imagery brain–computer interface using multimodal speech imagery. Journal of Medical and Biological Engineering, 43(3), 216-226.
151. Santhakumari, S., & Kamalakannan, J. (2024). A Review on Applications of Electroencephalogram: Includes Imagined Speech. International Journal of Advanced Computer Science & Applications, 15(2).
152. Lee, S. H., Lee, M., & Lee, S. W. (2021, February). Functional connectivity of imagined speech and visual imagery based on spectral dynamics. In 2021 9th International Winter Conference on Brain-Computer Interface (BCI) (pp. 1-6). IEEE.
153. Pei, X., Barbour, D. L., Leuthardt, E. C., & Schalk, G. (2011). Decoding vowels and consonants in spoken and imagined words using electrocorticographic signals in humans. Journal of neural engineering, 8(4), 046028.
154. Cooney, C., Korik, A., Raffaella, F., & Coyle, D. (2019, September). Classification of imagined spoken word-pairs using convolutional neural networks. In The 8th Graz BCI Conference, 2019 (pp. 338-343). Verlag der Technischen Universitat Graz.
155. Zhou, J., Duan, Y., Zou, Y., Chang, Y. C., Wang, Y. K., & Lin, C. T. (2023). Speech2EEG: Leveraging pretrained speech model for EEG signal recognition. IEEE Transactions on Neural Systems and Rehabilitation Engineering, 31, 2140-2153.
156. Wang, L., Huang, W., Yang, Z., & Zhang, C. (2020). Temporal-spatial-frequency depth extraction of brain-computer interface based on mental tasks. Biomedical Signal Processing and Control, 58, 101845.
157. Jahangiri, A., & Sepulveda, F. (2019). The relative contribution of high-gamma linguistic processing stages of word production, and motor imagery of articulation in class separability of covert speech tasks in EEG data. Journal of medical systems, 43(2), 20.
158. Bisla, M., & Anand, R. S. (2024). Optimized CNN‐Bi‐LSTM–Based BCI System for Imagined Speech Recognition Using FOA‐DWT. Advances in Human‐Computer Interaction, 2024(1), 8742261.
159. Tøttrup, L., Leerskov, K., Hadsund, J. T., Kamavuako, E. N., Kæseler, R. L., & Jochumsen, M. (2019, June). Decoding covert speech for intuitive control of brain-computer interfaces based on single-trial EEG: a feasibility study. In 2019 IEEE 16th International Conference on Rehabilitation Robotics (ICORR) (pp. 689-693). IEEE.
160. Moctezuma, L. A., Torres-García, A. A., Villaseñor-Pineda, L., & Carrillo, M. (2019). Subjects identification using EEG-recorded imagined speech. Expert Systems with Applications, 118, 201-208.
161. Cooney, C., Folli, R., & Coyle, D. (2019, October). Optimizing layers improves CNN generalization and transfer learning for imagined speech decoding from EEG. In 2019 IEEE international conference on systems, man and cybernetics (SMC) (pp. 1311-1316). IEEE.
162. Balaji, A., Haldar, A., Patil, K., Ruthvik, T. S., Ca, V., Jartarkar, M., & Baths, V. (2017, July). EEG-based classification of bilingual unspoken speech using ANN. In 2017 39th Annual International Conference of the IEEE Engineering in Medicine and Biology Society (EMBC) (pp. 1022-1025). IEEE.
163. Sarmiento, L. C., Villamizar, S., López, O., Collazos, A. C., Sarmiento, J., & Rodríguez, J. B. (2021). Recognition of EEG signals from imagined vowels using deep learning methods. Sensors, 21(19), 6503.
164. Bakhshali, M. A., Khademi, M., Ebrahimi-Moghadam, A., & Moghimi, S. (2020). EEG signal classification of imagined speech based on Riemannian distance of correntropy spectral density. Biomedical Signal Processing and Control, 59, 101899.
165. Zheng, X. B., Ling, B. W. K., Zheng, S. Y., & Li, C. J. (2023). Supervised categorized principal component analysis for imagined speech classification via applying singular value decomposition on a symmetry matrix. Biomedical Signal Processing and Control, 86, 105324.
166. Bisla, M., & Anand, R. S. (2023, April). EEG based brain computer interface system for decoding covert speech using deep neural networks. In 2023 IEEE 12th International Conference on Communication Systems and Network Technologies (CSNT) (pp. 414-419). IEEE.
167. Koizumi, K., Ueda, K., & Nakao, M. (2018, July). Development of a cognitive brain-machine interface based on a visual imagery method. In 2018 40th Annual International Conference of the IEEE Engineering in Medicine and Biology Society (EMBC) (pp. 1062-1065). IEEE.
168. Tsukahara, A., Yamada, M., Tanaka, K., & Uchikawa, Y. (2019, July). Analysis of eeg frequency components and an examination of electrodes localization during speech imagery. In 2019 41st Annual International Conference of the IEEE Engineering in Medicine and Biology Society (EMBC) (pp. 4698-4702). IEEE.
169. van den Berg, B., van Donkelaar, S., & Alimardani, M. (2021, September). Inner speech classification using EEG signals: A deep learning approach. In 2021 IEEE 2nd International Conference on Human-Machine Systems (ICHMS) (pp. 1-4). IEEE.
170. Martin, S., Iturrate, I., Millán, J. D. R., Knight, R. T., & Pasley, B. N. (2018). Decoding inner speech using electrocorticography: Progress and challenges toward a speech prosthesis. Frontiers in neuroscience, 12, 422.
171. Nieto, N., Peterson, V., Rufiner, H. L., Kamienkowski, J. E., & Spies, R. (2022). Thinking out loud, an open-access EEG-based BCI dataset for inner speech recognition. Scientific data, 9(1), 52.
172. Cooney, C., Korik, A., Folli, R., & Coyle, D. (2020). Evaluation of hyperparameter optimization in machine and deep learning methods for decoding imagined speech EEG. Sensors, 20(16), 4629.
173. Bakhshali, M. A., Khademi, M., & Ebrahimi-Moghadam, A. (2022). Investigating the neural correlates of imagined speech: An EEG-based connectivity analysis. Digital Signal Processing, 123, 103435.
174. Keles, T., Yildiz, A. M., Barua, P. D., Dogan, S., Baygin, M., Tuncer, T., ... & Acharya, U. R. (2023). A new one-dimensional testosterone pattern-based EEG sentence classification method. Engineering Applications of Artificial Intelligence, 119, 105722.
175. Hossain, A., Khan, P., & Kader, M. F. (2024). Imagined speech classification exploiting EEG power spectrum features. Medical & biological engineering & computing, 62(8), 2529-2544.
176. Carvalho, V. R., Mendes, E. M. A. M., Fallah, A., Sejnowski, T. J., Comstock, L., & Lainscsek, C. (2024). Decoding imagined speech with delay differential analysis. Frontiers in Human Neuroscience, 18, 1398065.
177. Mahapatra, N. C., & Bhuyan, P. (2022, November). Decoding of imagined speech neural EEG signals using deep reinforcement learning technique. In 2022 International Conference on Advancements in Smart, Secure and Intelligent Computing (ASSIC) (pp. 1-6). IEEE.
178. Al-Hammuri, K., Gebali, F., Thirumarai Chelvan, I., & Kanan, A. (2022). Tongue contour tracking and segmentation in lingual ultrasound for speech recognition: A review. Diagnostics, 12(11), 2811.
179. Soroush, P. Z., Angrick, M., Shih, J., Schultz, T., & Krusienski, D. J. (2021, October). Speech activity detection from stereotactic EEG. In 2021 IEEE International Conference on Systems, Man, and Cybernetics (SMC) (pp. 3402-3407). IEEE.
180. Ng, H. W., & Guan, C. (2024). Subject-independent meta-learning framework towards optimal training of EEG-based classifiers. Neural Networks, 172, 106108.
181. Huang, W., Zhao, J., & Fu, W. (2018). A deep learning approach based on CSP for EEG analysis. In Intelligent Information Processing IX: 10th IFIP TC 12 International Conference, IIP 2018, Nanning, China, October 19-22, 2018, Proceedings 10 (pp. 62-70). Springer International Publishing.
182. Brumberg, J. S., Burnison, J. D., & Pitt, K. M. (2016). Using motor imagery to control brain-computer interfaces for communication. In Foundations of Augmented Cognition: Neuroergonomics and Operational Neuroscience: 10th International Conference, AC 2016, Held as Part of HCI International 2016, Toronto, ON, Canada, July 17-22, 2016, Proceedings, Part I 10 (pp. 14-25). Springer International Publishing.
183. Kaongoen, N., Choi, J., & Jo, S. (2021, February). Transcranial Direct-Current Stimulation Effect on a Speech-Imagery-based BCI. In 2021 9th International Winter Conference on Brain-Computer Interface (BCI) (pp. 1-5). IEEE.
184. Padfield, N., Camilleri, T., Fabri, S., Bugeja, M., & Camilleri, K. (2024). A combined EEG motor and speech imagery paradigm with automated successive halving for customizable command selection. Brain-Computer Interfaces, 11(3), 125-142.
185. Biswas, S., & Sinha, R. (2022). Wavelet filterbank‐based EEG rhythm‐specific spatial features for covert speech classification. IET Signal Processing, 16(1), 92-105.
186. Sereshkeh, A. R., Yousefi, R., Wong, A. T., & Chau, T. (2018). Online classification of imagined speech using functional near-infrared spectroscopy signals. Journal of neural engineering, 16(1), 016005.
187. Manca, A. D., De Nunzio, G., & Grimaldi, M. (2016). EEG‐based recognition of silent and imagined vowels. Phonetics and Language Learning, Milano, 305-321.
188. Panachakel, J. T., Ramakrishnan, A. G., & Ananthapadmanabha, T. V. (2019, December). Decoding imagined speech using wavelet features and deep neural networks. In 2019 IEEE 16th India Council International Conference (INDICON) (pp. 1-4). IEEE.
189. Biswas, S., & Sinha, R. (2018, December). Lateralization of brain during EEG based covert speech classification. In 2018 15th IEEE India Council International Conference (INDICON) (pp. 1-5). IEEE.
190. Moon, J., & Chau, T. (2023). Shared and task-specific phase coding characteristics of gamma-and theta-bands in speech perception and covert speech. Speech Communication, 147, 63-73.
191. Li, F., Chao, W., Li, Y., Fu, B., Ji, Y., Wu, H., & Shi, G. (2021). Decoding imagined speech from EEG signals using hybrid-scale spatial-temporal dilated convolution network. Journal of neural engineering, 18(4), 0460c4.
192. González-Castañeda, E. F., Torres-García, A. A., Reyes-García, C. A., & Villaseñor-Pineda, L. (2017). Sonification and textification: Proposing methods for classifying unspoken words from EEG signals. Biomedical Signal Processing and Control, 37, 82-91.
193. Noreika, V., Canales-Johnson, A., Koh, J., Taylor, M., Massey, I., & Bekinschtein, T. A. (2015). Intrusions of a drowsy mind: neural markers of phenomenological unpredictability. Frontiers in psychology, 6, 202.
194. Cooney, C., Folli, R., & Coyle, D. (2021). A bimodal deep learning architecture for EEG-fNIRS decoding of overt and imagined speech. IEEE Transactions on Biomedical Engineering, 69(6), 1983-1994.
195. Rezazadeh Sereshkeh, A., Yousefi, R., Wong, A. T., Rudzicz, F., & Chau, T. (2019). Development of a ternary hybrid fNIRS-EEG brain–computer interface based on imagined speech. Brain-Computer Interfaces, 6(4), 128-140.
196. Kaongoen, N., Choi, J., & Jo, S. (2022). A novel online BCI system using speech imagery and ear-EEG for home appliances control. Computer methods and programs in biomedicine, 224, 107022.
197. García-Salinas, J. S., Villaseñor-Pineda, L., Reyes-García, C. A., & Torres-García, A. A. (2019). Transfer learning in imagined speech EEG-based BCIs. Biomedical Signal Processing and Control, 50, 151-157.
198. Kaongoen, N., Choi, J., & Jo, S. (2021). Speech-imagery-based brain–computer interface system using ear-EEG. Journal of neural engineering, 18(1), 016023.
199. Datta, S., & Boulgouris, N. V. (2021). Recognition of grammatical class of imagined words from EEG signals using convolutional neural network. Neurocomputing, 465, 301-309.
200. Li, M., Pun, S. H., & Chen, F. (2023, July). Impacts of cortical regions on EEG-based classification of lexical tones and vowels in spoken speech. In 2023 45th Annual International Conference of the IEEE Engineering in Medicine & Biology Society (EMBC) (pp. 1-4). IEEE.
201. Kamble, A., Ghare, P. H., Kumar, V., Kothari, A., & Keskar, A. G. (2023). Spectral analysis of EEG signals for automatic imagined speech recognition. IEEE Transactions on Instrumentation and Measurement, 72, 1-9.
202. Hashim, N., Ali, A., & Mohd-Isa, W. N. (2017, November). Word-based classification of imagined speech using EEG. In International Conference on Computational Science and Technology (pp. 195-204). Singapore: Springer Singapore.
203. Simistira Liwicki, F., Gupta, V., Saini, R., De, K., Abid, N., Rakesh, S., Wellington, S., Wilson, H., Liwicki, M., & Eriksson, J. (2023). Bimodal electroencephalography-functional magnetic resonance imaging dataset for inner-speech recognition. Scientific Data, 10(1), 378.
204. Kiroy, V. N., Bakhtin, O. M., Krivko, E. M., Lazurenko, D. M., Aslanyan, E. V., Shaposhnikov, D. G., & Shcherban, I. V. (2022). Spoken and inner speech-related EEG connectivity in different spatial direction. Biomedical Signal Processing and Control, 71, 103224.
205. Shah, U., Alzubaidi, M., Mohsen, F., Abd-Alrazaq, A., Alam, T., & Househ, M. (2022). The Role of Artificial Intelligence in Decoding Speech from EEG Signals: A Scoping Review. *Sensors*, *22*(18), 6975.
206. García-Salinas, J. S., Villaseñor-Pineda, L., Reyes-García, C. A., & Torres-García, A. (2018). Tensor decomposition for imagined speech discrimination in EEG. In Advances in Computational Intelligence: 17th Mexican International Conference on Artificial Intelligence, MICAI 2018, Guadalajara, Mexico, October 22–27, 2018, Proceedings, Part II 17 (pp. 239-249). Springer International Publishing.
207. Bisla, M., & Anand, R. S. (2024). Transfer learning enabled imagined speech interpretation using phase-based brain functional connectivity and power analysis. IEEE Access.
208. Yao, B., Taylor, J. R., Banks, B., & Kotz, S. A. (2021). Reading direct speech quotes increases theta phase-locking: Evidence for cortical tracking of inner speech?. NeuroImage, 239, 118313.
209. Villena-González, M., Palacios-García, I., Rodríguez, E., & López, V. (2018). Beta oscillations distinguish between two forms of mental imagery while gamma and theta activity reflects auditory attention. Frontiers in human neuroscience, 12, 389.
210. Juyal, R., Muthusamy, H., Kumar, N., & Tiwari, A. (2024). Resting state EEG assisted imagined vowel phonemes recognition by native and non-native speakers using brain connectivity measures. Physical and Engineering Sciences in Medicine, 47(3), 939-954.
211. Jeong, J. H., Cho, J. H., Lee, B. H., & Lee, S. W. (2022). Real-time deep neurolinguistic learning enhances noninvasive neural language decoding for brain–machine interaction. IEEE Transactions on Cybernetics, 53(12), 7469-7482.
212. Wu, S., Bhadra, K., Giraud, A. L., & Marchesotti, S. (2024). Adaptive LDA Classifier Enhances Real-Time Control of an EEG Brain–Computer Interface for Decoding Imagined Syllables. Brain Sciences, 14(3), 196.
213. Ji, Y., Li, F., Fu, B., Zhou, Y., Wu, H., Li, Y., Li, X., & Shi, G. (2024). A novel hybrid decoding neural network for EEG signal representation. Pattern Recognition, 155, 110726.
214. Wang, L., Yan, Z., Liu, Y., & Hu, L. (2021). Analysis and application of functional connectivity in synchronic hybrid mental tasks for brain-computer interface. Measurement, 186, 110116.
215. Moon, J., Orlandi, S., & Chau, T. (2022). A comparison and classification of oscillatory characteristics in speech perception and covert speech. Brain Research, 1781, 147778.
216. Assaneo, M. F., & Poeppel, D. (2018). The coupling between auditory and motor cortices is rate-restricted: Evidence for an intrinsic speech-motor rhythm. Science advances, 4(2), eaao3842.
217. Alderson-Day, B., Mitrenga, K., Wilkinson, S., McCarthy-Jones, S., & Fernyhough, C. (2018). The varieties of inner speech questionnaire–Revised (VISQ-R): Replicating and refining links between inner speech and psychopathology. Consciousness and cognition, 65, 48-58.
218. Varnet, L., Ortiz-Barajas, M. C., Erra, R. G., Gervain, J., & Lorenzi, C. (2017). A cross-linguistic study of speech modulation spectra. The Journal of the Acoustical Society of America, 142(4), 1976-1989.
219. Yao, B., Taylor, J. R., Banks, B., & Kotz, S. A. (2020). Theta activity phase-locks to inner speech in silent reading. PsyAr**v.
220. Kim, J., Park, Y., & Chung, W. (2020, February). Transform based feature construction utilizing magnitude and phase for convolutional neural network in EEG signal classification. In 2020 8th International Winter Conference on Brain-Computer Interface (BCI) (pp. 1-4). IEEE.
221. Boloukian, B., & Safi-Esfahani, F. (2020). Recognition of words from brain-generated signals of speech-impaired people: Application of autoencoders as a neural Turing machine controller in deep neural networks. Neural Networks, 121, 186-207.
222. Värbu, K., Muhammad, N., & Muhammad, Y. (2022). Past, present, and future of EEG-based BCI applications. Sensors, 22(9), 3331.
223. Herff, C., & Schultz, T. (2016). Automatic speech recognition from neural signals: a focused review. Frontiers in neuroscience, 10, 429.
224. Brumberg, J. S., Pitt, K. M., & Burnison, J. D. (2018). A noninvasive brain-computer interface for real-time speech synthesis: The importance of multimodal feedback. IEEE Transactions on Neural Systems and Rehabilitation Engineering, 26(4), 874-881.
225. Torres-García, A. A., Reyes-García, C. A., Villaseñor-Pineda, L., & García-Aguilar, G. (2016). Implementing a fuzzy inference system in a multi-objective EEG channel selection model for imagined speech classification. Expert Systems with Applications, 59, 1-12.
226. Akbari, H., Khalighinejad, B., Herrero, J. L., Mehta, A. D., & Mesgarani, N. (2019). Towards reconstructing intelligible speech from the human auditory cortex. Scientific reports, 9(1), 874.
227. Iliopoulos, A. C., & Papasotiriou, I. (2022). Functional complex networks based on operational architectonics: Application on eeg-based brain–computer interface for imagined speech. Neuroscience, 484, 98-118.
228. Zhang, G., Luo, J., Han, L., Lu, Z., Hua, R., Chen, J., & Che, W. (2021). A dynamic multi-scale network for EEG signal classification. Frontiers in Neuroscience, 14, 578255.
229. Whitford, T. J., Spencer, K. M., Godwin, M., Hirano, Y., Chung, L. K. H., Vodovozov, W., Griffiths, O., Harris, A. W. F., Pelley, M. E. L., & Jack, B. N. (2025). Gamma and theta/alpha-band oscillations in the electroencephalogram distinguish the content of inner speech. eneuro, 12(2).
230. Lawhern, V. J., Solon, A. J., Waytowich, N. R., Gordon, S. M., Hung, C. P., & Lance, B. J. (2018). EEGNet: a compact convolutional neural network for EEG-based brain–computer interfaces. Journal of neural engineering, 15(5), 056013.
231. Li, S., Zhu, H., & Tian, X. (2020). Corollary discharge versus efference copy: distinct neural signals in speech preparation differentially modulate auditory responses. Cerebral Cortex, 30(11), 5806-5820.
232. Asghari Bejestani, M. R., Mohammad Khani, G. R., Nafisi, V. R., & Darakeh, F. (2022). EEG‐Based Multiword Imagined Speech Classification for Persian Words. BioMed Research International, 2022(1), 8333084.
233. Tian, X., Zarate, J. M., & Poeppel, D. (2016). Mental imagery of speech implicates two mechanisms of perceptual reactivation. Cortex, 77, 1-12.
